# Enhancement of Photoemission on p-Type GaAs Using Surface Acoustic Waves

**DOI:** 10.3390/s20082419

**Published:** 2020-04-24

**Authors:** Boqun Dong, Andrei Afanasev, Rolland Johnson, Mona Zaghloul

**Affiliations:** 1 School of Engineering and Applied Science, The George Washington University, Washington, DC 20052, USA; 2 Department of Physics, The George Washington University, Washington, DC 20052, USA; 3 MuPlus, Inc., Newport News, VA 23606, USA

**Keywords:** surface acoustic waves, photoemission, quantum efficiency, piezoelectric, photocathode, GaAs, ZnO, recombination, electron lifetime

## Abstract

We demonstrate that photoemission properties of p-type GaAs can be altered by surface acoustic waves (SAWs) generated on the GaAs surface due to dynamical piezoelectric fields of SAWs. Multiphysics simulations indicate that charge-carrier recombination is greatly reduced, and electron effective lifetime in p-doped GaAs may increase by a factor of 10× to 20×. It implies a significant increase, by a factor of 2× to 3×, of quantum efficiency (QE) for GaAs photoemission applications, like GaAs photocathodes. Conditions of different SAW wavelengths, swept SAW intensities, and varied incident photon energies were investigated. Essential steps in SAW device fabrication on a GaAs substrate are demonstrated, including deposition of an additional layer of ZnO for piezoelectric effect enhancement, measurements of current–voltage (I–V) characteristics of the SAW device, and ability to survive high-temperature annealing. Results obtained and reported in this study provide the potential and basis for future studies on building SAW-enhanced photocathodes, as well as other GaAs photoelectric applications.

## 1. Introduction

**Figure 1:**
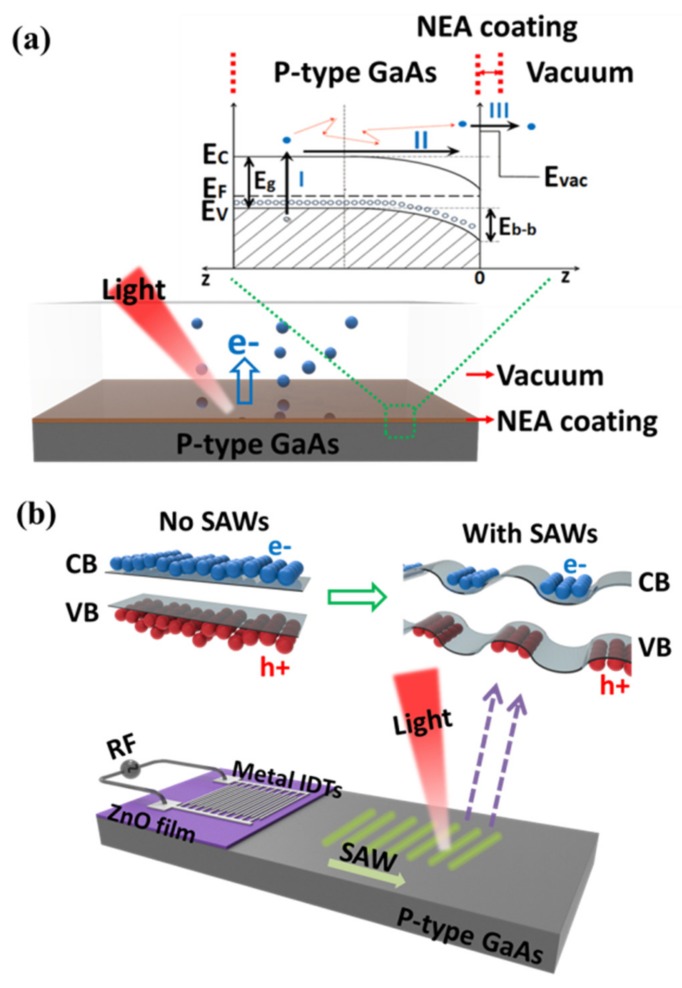
(**a**) The bottom is a three-dimensional (3D) schematic view showing the photoemission from p-type doped GaAs surface with a thin negative electron affinity (NEA) coating. The inset is a band diagram demonstration of the three-step photoemission mechanism: I. photoexcitation, II. transport, III. emission from the surface. (**b**) The 3D schematic view at bottom shows the concept and structure of our surface acoustic wave (SAW) device used to generate SAWs on p-type doped GaAs substrate. Top left—the result of photoexcitation without SAWs. Top right—the band bending effect caused by SAWs. In this case, the electrons and holes are spatially separated, thus the recombination is suppressed.

Photocathodes are used as a source of electrons for numerous applications that include photomultipliers, electron microscopes, and particles accelerators [1,2,3]. If spin-polarized electrons are required for applications, a standard choice are III–V semiconductors activated to negative electron affinity (NEA) coatings [4]. The three-step photoemission mechanism [5] of a p-type GaAs photocathode with NEA coating is shown in Figure 1a. The photovoltaic effect and semiconductor properties of GaAs are utilized to achieve photoexcitation (step I) and electron transportation (step II). The NEA coating is used to lower the vacuum level to below the conduction band minimum for electron emission (step III) [5]. Note that defects existing in GaAs will increase the density of surface states inside the forbidden gap, which leads to a slight shift in the photoemission spectra [6]. High quantum efficiency (QE) of photocathodes is an important requirement, for example, for an electron-ion collider for next-generation fundamental research in nuclear physics [7], where spin-polarized electron beams are needed to achieve research objectives. Recent progress [8] for increasing the QE of a spin-polarized GaAs/GaAsP superlattice electron source was due to the use of a distributed Bragg reflector that was designed to increase the photon absorption in a thin active layer of the photocathode.

In this work, we study the effect of piezoelectric fields on the minority carriers in p-doped GaAs structures. This research was motivated by findings [9] that piezoelectric fields generated in GaAs by surface acoustic waves (SAWs) suppress recombination and lead to extended electron lifetimes in this system. This is achieved by using SAWs to periodically bend the energy bands and to spatially separate electrons and holes [9,10,11] as shown in Figure 1b. Two of us [12] suggested earlier that suppression of charge-carrier recombination due to SAW may improve the performance of GaAs photocathodes.

In this present work, we use COMSOL Multiphysics as a tool to build models and to simulate step I and step II of the photoemission mechanism. The QE of the emission process in step III is derived based on theoretical principles. All the work is completed for both a bulk GaAs structure and a thin film GaAs structure.

## 2. Results and Discussion

### 2.1. Bulk p-Type GaAs

**Figure 2:**
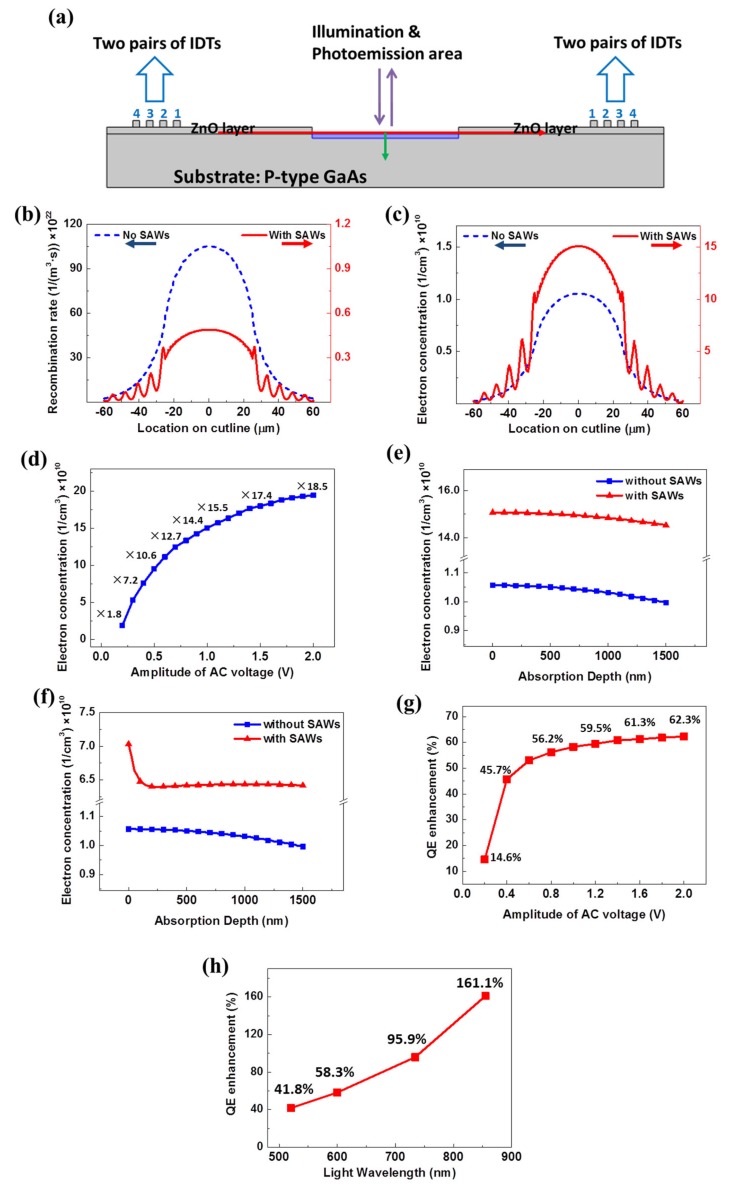
(**a**) Simulation structure of bulk p-type doped GaAs with interdigital transducers (IDTs). (**b**) Comparison of recombination rates with and without SAWs (*V_0_* = 1.0 V). (**c**) Comparison of surface electron concentrations with and without SAWs (*V_0_* = 1.0 V). (**d**) Increase of electron concentrations vs. AC voltage and SAW intensity. (**e**) Electron concentrations along absorption depth for SAW wavelength (λ_SAW_ = 9.2 μm) that was larger than the absorption depth (1.5 μm) in bulk GaAs. (**f**) Same as (**e**), but for λ_SAW_ = 0.5 μm, which was smaller than the absorption depth. (**g**) Enhancement of quantum efficiency (QE) under different amplitudes of AC voltage. (**h**) Enhancement of QE under different incident light wavelength (*V_0_* = 1.0 V).

Figure 2a shows the cross-section view of the simulation structure, which is the same as the 3D model shown in Figure 1b. The substrate was highly doped p-type GaAs with doping concentration of 5 × 10^18^ cm^−3^. A ZnO film (thickness: 2.0 μm) was placed on top of the GaAs to enhance the generation of SAWs due to its strong piezoelectric effect. This technique of using ZnO was studied and verified in our previous research [13]. Note that ZnO in the center region was removed to expose the GaAs surface for illumination and photoemission, and for the deposition of NEA coating in future study. Two pairs of interdigital transducers (IDTs) made of aluminum were deposited on top of the ZnO layer on each side of the illumination area and used to generate SAWs. IDTs 1 and 3 were grounded. A radio frequency AC voltage was applied to IDTs 2 and 4 that is expressed by the following equation:

(1) $$V_{in}=V_{0}\times sin\left(2\pi \times f_{0}\times t\right),$$

where *V_0_* was chosen as 1.0 V and *f_0_* was set to 300 MHz during simulation. The SAW was treated as a Rayleigh wave in the simulation. The wave propagating speed was set to 2760 m/s. Thus, the wavelength of SAW was calculated to be 9.2 μm. The thickness of substrate was 18.5 μm and the width was 190 μm. The IDTs were each 2.3 μm wide × 2.3 μm high. The distance between each IDT was also 2.3 μm.

In simulation, light illumination was applied to the center of the top of the bulk GaAs, as indicated in the purple rectangular area in Figure 2a. The width of the area was 50 μm and the depth was 1.5 μm. Photoexcited electrons were created under illumination by a laser beam. The power of the laser was set to 5 W/mm^2^. The wavelength of incident photons was set to 600 nm, thus the photon energy is 2.07 eV.

The results shown in Figure 2b,c were calculated and extracted along the red cutline indicated in Figure 2a. The cutline on the top surface of the highly p-doped GaAs was in the horizontal direction, which was also the propagation direction of the SAW. Results show that recombination rates were reduced by 10^2^–10^3^ times and electron concentrations increased about 14 times at the surface of the photoemission area when SAWs propagate along the surface of the GaAs.

In order to verify the dependence of the enhancement of the electron concentration on SAW intensity, the amplitude of the applied AC voltage was swept from 0.2 to 2.0 V in steps of 0.1 V. Simulation results are shown in Figure 2d. The y-axis shows the electron concentration at the GaAs surface with the effect of SAWs. The numbers indicated above the curve refer to different enhancements of surface electron concentrations with varied SAW intensities. With the increase of amplitude of AC voltage and thus the SAW intensity, more photoexcited electrons were able to reach the top surface of the GaAs. The maximum applied voltage *V_0_* in simulation was 2.0 V. A description of this voltage limit is given in Section 3.

Results in Figure 2e were calculated and extracted along the green cutline that is the photon absorption depth in GaAs in the vertical direction, as indicated in Figure 2a. In Figure 2e, the leftmost side (0 nm) of the x-axis refers to the top surface of GaAs, and the rightmost side (1500 nm) refers to the bottom of the absorption area. Results showed a stable and significant enhancement from the surface to the bottom of the absorption area since the wavelength of the SAW (9.2 μm) was larger than the depth of the absorption area (1.5 μm) in this simulation.

In order to investigate the dependence of electron concentration enhancement on the SAW wavelength, another simulation structure was modeled and calculated. In this model, the size of IDTs was greatly reduced to make the SAW wavelength (0.5 μm) smaller than the absorption depth (1.5 μm). This simulation result is shown in Figure 2f. The blue line is same as the one shown in Figure 2e, indicating the electron concentrations without SAWs. The trend of the red line, however, changed in this new simulation. The SAW enhancement was reduced after the absorption depth exceeded half the SAW wavelength.

According to the simulation results, that recombination was suppressed and electron concentration increased, the lifetime τ of electrons was increased by the same multiple based on the following equation:

(2) $$\frac{n_{\mathrm{SAW}}}{n}=\frac{\tau_{\mathrm{SAW}}}{\tau },$$

that follows from the relation between the equilibrium concentration of excess carriers (*n*), carrier lifetime (τ), and photoinjection rate (*r_in_*): $n=\tau \cdot r_{in}$. Next, based on:

(3) $$L=\sqrt{\tau \times D},$$

the diffusion length *L* extends with carrier lifetime τ [14].

The QE can be described by the Spicer’s three-step model of photoemission [15]:

(4) $$QE=\left(1-R\right)(\frac{\alpha_{PE}}{\alpha })(\frac{P_{E}}{1+\frac{l_{\alpha }}{L}}),$$

where *R* is the reflectivity, $\alpha_{PE}/\alpha$ is the fraction of electrons excited above vacuum level, $l_{\alpha }$ is the absorption depth, *L* is the diffusion length, and *P_E_* is the probability that electrons penetrate the NEA barrier and escape into the vacuum, after reaching the sample’s surface. For this bulk GaAs model, the photoexcited electron was assumed to have only one chance to hit the surface for photoemission. Hence, the diffusion length *L* is the only parameter in this formula that will change with the application of SAWs, and thus the SAW-enhanced QE can be derived by the equation:

(5) $$\frac{QE_{SAW}}{QE_{0}}=\frac{1+\frac{l_{\alpha }}{L_{0}}}{1+\frac{l_{\alpha }}{L_{SAW}}},$$

For the calculation, *L_0_* was set to 1.5 μm according to literature [14]. The variable $l_{\alpha }$ was 1.5 μm as used in the simulation. Based on the increased electron concentrations shown in Figure 2d, *L_SAW_* was calculated using Equations (2) and (3). Then the QE enhancement ($QE_{SAW}/QE_{0}-1$) for varied amplitudes of AC voltage was calculated and shown in Figure 2g. 

According to the literature [16], the absorption depth in GaAs is dependent on the wavelength of incident photons. To evaluate the QE improvement as a function of the light wavelength, we used different absorption depths corresponding to varied incident photon energies, which are 1.45 eV (855 nm), 8 um; 1.69 eV (734 nm), 3 um; 2.07 eV (600 nm), 1.5 um; 2.39 eV (520 nm), 1 um [16]. In this set of simulations, all other parameters including the structure of IDTs and bulk p-type GaAs model, the parameters of the SAW, and the applied AC voltage were the same as those described in Figure 2a. The simulation and calculation result of the QE enhancement due to the SAW is shown in Figure 2h. According to the result, the effect in bulk GaAs was strongly pronounced for $l_{\alpha }\gg L$, i.e., for photon energies approaching the band-gap, leading to enhanced QE for the photons of longer wavelengths.

### 2.2. Thin Film p-Type GaAs

**Figure 3:**
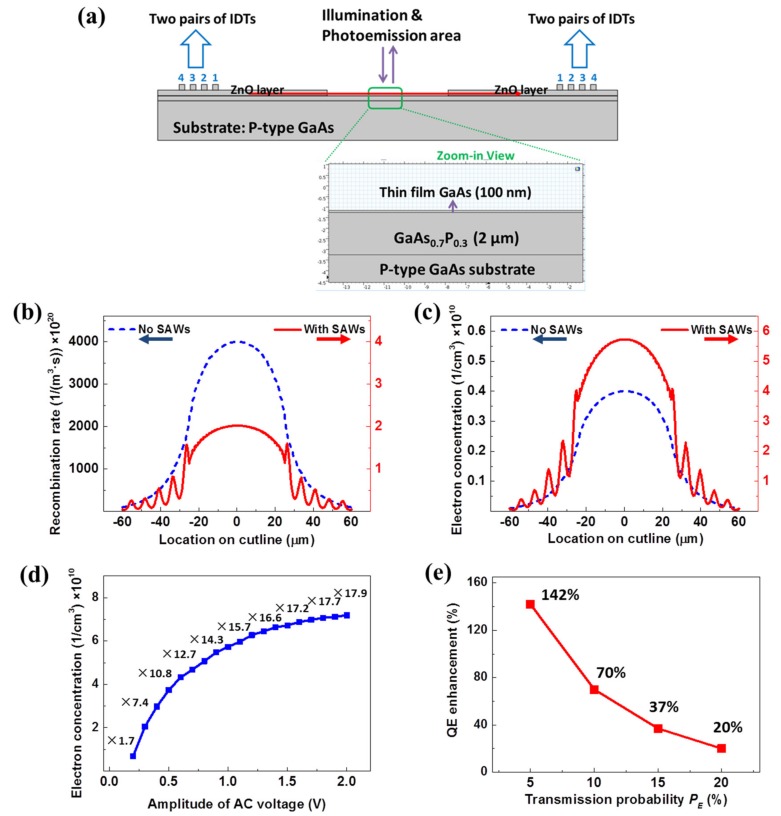
(**a**) Simulation structure of thin film GaAs with IDTs. (**b**) Comparison of recombination rates in GaAs thin film with and without SAWs (*V_0_* = 1.0 V). (**c**) Comparison of surface electron concentrations with and without SAWs (*V_0_* = 1.0 V). (**d**) Increase of electron concentrations caused by SAWs for different amplitudes of AC voltage. (**e**) Enhancement of QE caused by SAWs under different transmission probability *P_E_*.

In this section, the structure of a thin film of highly doped p-type GaAs placed on top surface was modeled and simulated. As shown in Figure 3a, the substrate was p-type GaAs with a doping concentration of 1 × 10^18^ cm^−3^. A layer of GaAs_0.7_P_0.3_ (thickness: 2.0 μm) was placed on GaAs substrate to create a potential barrier between surface and substrate. Then a thin layer of p-type GaAs was placed on top of GaAs_0.7_P_0.3_. The thickness was 100 nm and the doping concentration was 5 × 10^18^ cm^−3^. The structure of IDTs and ZnO film (center region was removed), the parameters of SAWs, and the applied AC voltage were the same as those used for the bulk p-type GaAs model (Figure 2a).

In this simulation, illumination was applied to the thin film of p-doped GaAs. The power of the laser beam was 5 W/mm^2^. The wavelength of incident photons was set to 800 nm, thus the photon energy was 1.55 eV. Corresponding simulation results were calculated along the red cutline (in Figure 3a), which refers to the propagation direction of the SAW on the top surface of the thin film GaAs. Results are plotted in Figure 3b–d. Same as in the bulk GaAs simulation, the recombination was greatly suppressed by 10^3^ times because of the SAWs. The amount of electron concentration at the surface was lower compared with the results shown in Figure 2c,d because the absorption area of thin film GaAs was smaller than that of bulk GaAs. However, the electron concentrations were still increased more than ten times due to SAWs.

Figure 3d shows the dependence of electron concentration enhancement on SAW intensity. The x-axis is the amplitude of applied AC voltage swept from 0.2 to 2.0 V. The y-axis shows the electron concentration at the surface of the p-doped GaAs thin film under the effect of SAWs. The numbers indicated above the curve refer to different enhancements of surface electron concentrations with varied SAW intensities. According to the result, a higher SAW intensity led to a larger enhancement.

For the thin film GaAs model, the photoexcited electron had multiple chances *N* to hit the surface for photoemission because of two reasons: first, *d* << *L*; second, the potential barrier created by the GaAs_0.7_P_0.3_ layer helped to push back electrons for another try. Therefore, according to Equation (4), the SAW-enhanced QE can be written in the following equations:

(6) $$\frac{QE_{SAW}}{QE_{0}}=\frac{1-{\left(1-P_{E}\right)}^{N_{SAW}}}{1-{\left(1-P_{E}\right)}^{N_{0}}},$$

(7) $$N=\frac{L}{2\times d}+1,$$

For the calculation, *d* was 0.1 μm as used in the simulation, *L_0_* was 1.5 μm according to literature [14], and *L_SAW_* was 6.4 μm as calculated using Equations (2) and (3) based on the result (at *V_0_* = 2.0 V) shown in Figure 3d. By sweeping *P_E_* from 5% to 20%, the QE enhancement ($QE_{SAW}/QE_{0}-1$) was calculated and plotted in Figure 3e. Results show that more dramatic enhancement from SAWs was achieved for cases of smaller transmission probability *P_E_*.

## 3. Fabrication and Experimentation of the SAW Device

**Figure 4:**
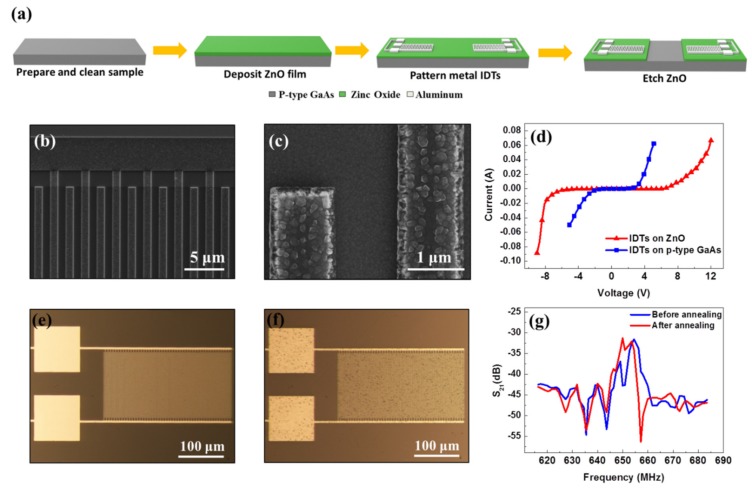
(**a**) Fabrication process flow of the device: deposition of ZnO thin layer, e-beam lithography and e-beam metal evaporation for aluminum IDTs, etch ZnO to open a window for exposing GaAs surface in center. (**b**) Scanning electron microscope (SEM) image of the top surface view of IDT fingers placed on the ZnO film. (**c**) High-resolution SEM image showing the surface morphology of the aluminum IDT finger and the c-axis oriented ZnO layer. (**d**) Current–voltage (I–V) characteristics of IDTs fabricated on different materials. (**e**) Optical microscope images of the IDTs taken before annealing. (**f**) Optical microscope images of the IDTs taken after annealing. (**g**) Comparison of transmission property S_21_ of SAWs generated before and after annealing.

Next, to fabricate and characterize the SAW device, we used highly doped p-type GaAs as substrate. After preparing the sample with a standard cleaning process, ZnO thin layer was deposited on the GaAs substrate using an RF magnetron sputtering system with an optimized recipe. After spin-coating photoresist on top of ZnO layer, e-beam lithography technique was used for transferring the pattern on the photoresist. After developing the patterned photoresist, aluminum was deposited as metal IDTs using an e-beam evaporator. Lastly, the center region of the ZnO layer was etched and removed so that the surface of GaAs substrate was not covered by ZnO. This allowed for the deposition of NEA coating on the GaAs surface for a photoemission experiment in a future study. The fabrication process is shown in Figure 4a. The fabricated devices were characterized by optical microscope and scanning electron microscope (SEM) to make sure the device structure, dimensions, and surface morphology were achieved as designed.

Figure 4b,c present the outcome of the IDT device fabrication on a ZnO film and highly p-doped GaAs substrate, clearly showing the metal IDT fingers were well shaped. The metal surface was a little rough due to the quality of chamber vacuum during the e-beam evaporation process. The ZnO surface, however, was smooth and clean as needed for generating SAWs.

We used a semiconductor device analyzer (Agilent B1500A) to measure the current–voltage (I–V) characteristics of the IDT device. The test sample was placed on a probe station (Signatone 1160 Series) and was connected to two DC probes. Each DC probe had one pin. One probe was placed on the ground contact pad and the other probe was placed on the source contact pad. On the other end, the two DC probes were connected to the semiconductor device analyzer via two BNC-to-alligator cables. Then the scanning DC voltage was applied between the IDT fingers and the corresponding current was measured using the semiconductor device analyzer.

Figure 4d shows two measured I–V curves of the IDT devices fabricated on p-type GaAs substrate with and without the ZnO film. Results showed that the IDT directly deposited on the GaAs substrate had a safety limit of 2.5 volts. Above this limit, the current increased rapidly and the device was in danger of being damaged. Next, we can see the voltage limit of the device with the ZnO film increased from 2 to 6 volts. This was because ZnO has larger energy band-gap than GaAs, thus it is able to resist a higher voltage and is more difficult to break down.

Additionally, since many photoemission applications such as photocathodes have heat processing during operation [17], we put our device into an annealing system (500 °C, 10 min) to test its robustness. By comparing Figure 4e,f, it is clear that both the metal IDTs and the surface of ZnO film were not damaged during the annealing process. The only change was that some small dark spots appeared on the surface after annealing. Most of the dots were small dirt and defects that were originally located on surface and were exposed after annealing, thus did not reduce the performance of the SAW.

To test the performance of generated SAWs, a network analyzer (Agilent 8722D) was used to apply scanning radio frequency (RF) signals between input IDT fingers, and to measure the transmission characteristics S_21_ of SAWs at the output IDT. The device was placed on the probe station. In this case, both input IDT and output IDT were connected to an RF probe, separately. Next, the two RF probes were connected to the two ports of the network analyzer via two transmission line cables. The test was conducted on the same sample before and after annealing. As shown in Figure 4g, the peak value and bandwidth of the transmission coefficient S_21_ were consistent before and after annealing, proving the SAW was still able to be generated and was transmitted successfully after the high-temperature annealing process. As described at the beginning of Section 2.1, one of the key points in this design was to have the SAW propagating along the uncovered surface of highly doped p-type GaAs, which was a challenge because the piezoelectric coupling coefficient of GaAs is much smaller compared to those of the commonly used piezoelectric materials [18,19]. Thus, we developed the technique of using ZnO islands to enhance the generation and propagation of SAWs on highly doped GaAs. This technique improved and provided a much better transmission performance S_21_ of SAWs (Figure 4g) on uncovered p-type GaAs, although it was not as perfect as those obtained on traditional piezoelectric materials. Detailed information and description of this technique and the corresponding experiments were presented in our previous report [13]. 

## 4. Applications

The described method to increase the QE of photoemission on p-type GaAs is the essential step to improve the performance of photomultipliers, the extremely sensitive light detectors in the ultraviolet, visible light, and near-infrared ranges. With the SAW-enhanced design, the spectral range of photomultipliers can be improved, especially the sensitivity for the photon energies approaching the semiconductor’s band gap.

In addition, using the demonstrated technique to suppress the recombination and increase carriers’ lifetime in highly doped GaAs is also important and beneficial to enhance other types of photosensors, such as the photodiode-based III–V semiconductor photodetectors, for which more photoexcited carriers can be collected and expected to generate a higher photocurrent.

Other important applications are expected for further study based on the SAW-enhanced photoemission design, namely: 

High-brightness unpolarized electron sources for high-energy physics, whereby increasing QE without changing the laser beam spot size on a photocathode surface provides higher beam brightness; 

Polarized electron sources for particle accelerators [3], where the proposed method of modifying electron dynamics can be combined with a distributed Bragg reflector method to increase light absorption [8] for high-intensity low-emittance beams for fundamental nuclear physics research [7]; 

Electron microscopy, for which electron beam emittance can be reduced for the same beam intensity due to reduced laser-spot size.

## 5. Conclusions

In summary, we have demonstrated via simulations that SAWs applied to p-doped GaAs extend the minority-carrier (electron) lifetimes by over an order of magnitude. Importantly, for the applications in spin-polarized electron sources, the extension of electron lifetimes is accompanied by extension of their polarization lifetimes, as seen in an experiment [20]. Results of different SAW wavelengths, swept SAW intensities, and varied photon energies are presented. Further calculations predict that the QE of GaAs photocathodes increase by a factor of 2 to 3, depending on the device geometry and the NEA-coating properties. In particular, reduction of the thin-film photocathode operational lifetime can be potentially mitigated by the SAW-enhanced electron lifetimes. Essential components of the SAW device were fabricated and showed high-quality SAW generation with an additional layer of ZnO applied under IDTs. We measured the device’s I–V characteristics, and its performance after high-temperature annealing. The findings and outcomes reported in this paper provide a good basis and guidance for future studies on designing and building SAW-enhanced GaAs photocathodes, photomultipliers, and other III–V semiconductor photoelectric applications.
